# Super-Resolution Microscopy Reveals an Altered Fibrin Network in Cirrhosis: The Key Role of Oxidative Stress in Fibrinogen Structural Modifications

**DOI:** 10.3390/antiox9080737

**Published:** 2020-08-12

**Authors:** Matteo Becatti, Amanda Mannucci, Flavia Rita Argento, Stefano Gitto, Francesco Vizzutti, Fabio Marra, Niccolò Taddei, Claudia Fiorillo, Giacomo Laffi

**Affiliations:** 1 Department of Experimental and Clinical Biomedical Sciences “Mario Serio”, University of Florence, Viale Morgagni 50, 50134 Florence, Italy; amanda.mannucci@unifi.it (A.M.); flaviarita.argento@unifi.it (F.R.A.); niccolo.taddei@unifi.it (N.T.); claudia.fiorillo@unifi.it (C.F.); 2 Department of Experimental and Clinical Medicine, University of Florence, Largo Brambilla 3, 50134 Florence, Italy; stefano.gitto@unifi.it (S.G.); francesco.vizzutti@unifi.it (F.V.); fabio.marra@unifi.it (F.M.); giacomo.laffi@unifi.it (G.L.)

**Keywords:** fibrinogen oxidation, fibrin structure, stimulated emission depletion (STED) microscopy, thrombus, cirrhosis

## Abstract

Cirrhotic patients show a reduced synthesis of both pro- and anti-coagulant factors. Recent reports indicate that they are characterized by a higher risk of thrombotic rather than hemorrhagic complications, but the mechanisms conferring this risk are not fully elucidated. Oxidative-mediated fibrinogen modifications may explain, at least in part, a prothrombotic profile. The aim of the present pilot study was to investigate the alterations in fibrinogen structure and function in patients with cirrhosis of various severity and to correlate these findings with the mechanisms of thrombus formation. We assessed in plasma specific oxidative stress markers and measured oxidative modifications, functional and structural parameters in purified fibrinogen fractions obtained from cirrhotic patients and control subjects. We enrolled 15 cirrhotic patients (5 patients belonging to each of the three Child–Turcotte–Pugh classes) and 20 age- and sex-matched healthy controls. Plasma redox status, fibrinogen oxidative modifications, thrombin-catalyzed fibrin polymerization and fibrin resistance to plasmin-induced lysis were significantly altered in cirrhotic patients and were associated to disease severity. Importantly, clot structure obtained by stimulated emission depletion (STED) super-resolution microscopy indicated modifications in fiber diameter and in clot porosity in cirrhotic patients. Fibrin fiber diameter significantly decreased in cirrhotic patients when compared to controls, and this difference became more marked with disease progression. In parallel, fibrin pore size progressively decreased along with disease severity. In cirrhotic patients, fibrinogen clot analysis and oxidative-dependent changes reveal novel structural and functional fibrinogen modifications which may favor thrombotic complications in cirrhosis.

## 1. Introduction

Cirrhosis is associated with complex changes in the hemostatic system. Since the liver synthesizes the majority of coagulation factors, for a long time cirrhosis-associated coagulopathy has been considered a major contributor to bleeding complications [1]. According to current concepts, in these patients the decreased levels of coagulation factors are associated with a simultaneous decrease in the levels of anticoagulant molecules. This “rebalanced hemostasis” is particularly unstable and explains the occurrence of both bleeding and thrombotic complications [2,3]. Indeed, both prospective [4] and retrospective [5] studies suggest that cirrhotic patients show an increased risk of deep vein thrombosis (DVT) and pulmonary embolism (PE), although the pathophysiological mechanisms of this susceptibility have not yet been elucidated [6,7]. Abnormalities in coagulation factors, slowing of portal blood flow, and endothelial injury may all contribute to thrombosis development. In turn, several elements, including bacterial translocation, stage of disease, and severity of portal hypertension, may contribute to the development of endothelial dysfunction [8].

Fibrinogen, a trimeric 340-kDa glycoprotein primarily synthesized in hepatocytes, has a prominent role in clot development and in particular in platelet aggregation and in the formation of the fibrin network Recent evidence indicates, in several pathologic contexts, that oxidative stress contributes to generating altered clot structures [9,10]. Importantly, oxidative stress has been implicated as a key contributor of several hepatic disorders, including alcoholic and nonalcoholic steatohepatitis, iron overload, and fibrogenesis [11]. Recent experimental data from our group have revealed that ROS-mediated modifications of the fibrinogen structure reflect alteration in its biological activity. Indeed, fibrinogen oxidation contributes to the formation of a more thrombogenic clot, mainly consisting of a tight fibrin network composed of filaments which are resistant to plasmin-induced lysis and with slightly decreased fiber size [12,13]. This deeply modified fibrin network can significantly contribute to vascular occlusion and thrombus development [14,15,16].

In this pilot study, we investigated the possible relationship between fibrinogen structure and function in patients with cirrhosis of various severity, to elucidate the mechanisms of thrombus formation and provide additional mechanistic information on the pro-thrombotic features of this condition.

## 2. Material and Methods

### 2.1. Patients

For this pilot study, we consecutively enrolled cirrhotic patients referred to our unit. Diagnosis of cirrhosis was based on the combination of patient’s history, physical examination, liver ultrasound, transient elastography, laboratory findings, endoscopy, and, when not contraindicated, liver biopsy. Ascites was detected by physical examination and confirmed by ultrasound. Exclusion criteria were as follows: the inability to express an informed and fully aware consent, less than 6 months’ abstinence from alcohol use, drugs interfering with the hemostatic system, cardiovascular renal/pulmonary diseases, diabetes mellitus, hepato-renal syndrome, spontaneous bacterial peritonitis, infection in the previous three months, portal vein thrombosis, and presence of malignancies or gastrointestinal bleeding in the previous three months. Child–Turcotte–Pugh [17] and MELD [18] scores were used in the classification of cirrhosis severity. In all patients with ascites, diagnostic paracentesis with the determination of ascitic PMN cell count was performed to exclude spontaneous bacterial peritonitis.

We also enrolled a control group composed by age- and sex-matched healthy volunteers. The study conformed to the principles outlined in the Declaration of Helsinki. Local Ethical Committee approved the study (date of approval 17 of April 2018; registry code 11306_bio). All participants gave their written informed consent before entering the study.

### 2.2. Sample Collection

Vacutainer tubes containing 0.109 mol/L buffered trisodium citrate (1:10) or EDTA (0.17 mol/L) were used for blood collection. After centrifugation (1500× *g* for 15 min at 4 °C), aliquots of plasma were used for experiments or stored at 80 °C for further analysis. Sodium citrate plasma was used immediately for fibrinogen purification.

### 2.3. Total Antioxidant Capacity (TAC) Assay

The ORAC method (oxygen radical absorbance capacity) was performed on plasma samples as previously described [19].

### 2.4. Plasma Lipid Peroxidation Estimation

Plasma lipid peroxidation levels were measured using the ALDetect Lipid Peroxidation Assay Kit (Enzo Life Sciences Inc.) following the manufacturer’s protocol.

### 2.5. Fibrinogen Purification

Fibrinogen was purified from patients and controls using the previously described ethanol precipitation method [14]. Fibrinogen concentration was determined spectrophotometrically at 280 nm (the extinction coefficient 1.51 mg/mL was used). Fibrinogen purity, assessed by densitometry of Coomassie-stained polyacrylamide gels after electrophoresis under reducing condition and expressed as a percentage of total protein content, resulted in 98.7 ± 1.9% of total protein content in controls and 97.5 ± 2.2% in patients with liver cirrhosis. No significant statistical difference was observed in the purification yield between controls and patients.

### 2.6. Protein Concentration Assay

Protein concentration was determined using the Bradford assay as previously described [15].

### 2.7. Protein Carbonyl (PC) Determination

Oxidative modification on plasma proteins and purified fibrinogen was assessed as previously reported [20]. The results, expressed in terms of nmol/mL of protein carbonyl (PC), were then normalized for protein concentration.

### 2.8. Fibrin Digestion with Plasmin and Electrophoretic Analysis of Plasmin Digests

Fibrin clots were prepared incubating fibrinogen (2 mg/mL final concentration) with thrombin (12 U/mL final concentration) in 20 μL of 100 mmol/L Tris/HCl, 5 mmol/L CaCl_2_, pH 7.4, for 1 h at 25 °C. Then, fibrin clots were digested with plasmin (5 μL of 100 μg/mL) for 6 h at 37 °C, as previously described [15,16]. The same lots of thrombin and of plasmin were used for all experiments. Then, aliquots from each fibrin digest (10 μg of fibrin) were loaded onto 4% to 12% Bis-Tris gels. After electrophoresis, gels were stained with Coomassie blue and band intensities were quantified by densitometry using Quantity-One software (Bio-Rad, Milan, Italy). Data were expressed as the ratio between the densitometric reading of the purified protein at 6 h of plasmin digestion and that of the undigested protein (time 0 for incubation with plasmin).

### 2.9. Thrombin-Catalyzed Fibrin Polymerization Assays

For functional analysis, purified fibrinogen fractions stored at −80 °C and not previously thawed were used. Fibrin polymerization was monitored at 405 nm in a 96-well micro titer plate reader (Synergy H1 Hybrid Multi-Mode Reader, BioTek Instruments Inc., Winooski, VT, USA) at 25 °C, as previously described [15,16]. Before the polymerization assay, control and patient fibrinogen samples were extensively dialyzed against 100 mmol/L Tris/HCl buffer, pH 7.4, and diluted to a final concentration of 1 mg/mL. To each reaction (in triplicate), 240 μL of fibrinogen (1 mg/mL) in 100 mmol/L Tris/HCl, 5 mmol/L CaCl_2_, pH7.4 were added. The polymerization reaction was started by adding 60 μL thrombin (at a final concentration of 0.25 U/mL). Absorbance was monitored for 160 min at 25 °C.

Absorbance curves were characterized using the following parameters: (1) the maximum slope (Vmax), calculated as the slope of the steepest part of the polymerization curve, which represents the rate of lateral protofibril association; (2) the lag phase, measured as the time elapsed until an increase in absorbance was seen, which reflects the time to the start of lateral fibril aggregation; (3) maximum absorbance (MaxAbs) of the growing clot, recorded 160 min after polymerization was initiated, which reflects an average fibrin fiber size and the number of protofibrils per fiber.

### 2.10. Circular Dichroism Spectra of Purified Fibrinogen Extracts

For Circular dichroism (CD) spectra, a protein concentration of 1 mg/mL was used. Spectra were recorded at 25 °C in 0.2 cm quartz cells from 250 to 195 nm (far-UV). Samples were filtered through 0.22 μmol/L filters and 5 spectra recorded for each sample. Molar ellipticity values [q] were calculated according to the following equation: [θ](deg−cm^2^dmol^−1^) = [θ(MRW)]/[10(*l*)(*c*)], where θ is the displacement from the baseline value *X* to the full range in degrees; MRW is the mean residue weight of the amino acids; (*l*) is the path length of the cell (cm); and (*c*) is protein concentration (g/mL) [21].

### 2.11. Intrinsic Fluorescence Spectra of Fibrinogen

Fibrinogen intrinsic fluorescence spectra were recorded at a protein concentration of 0.1 mg/mL and 25  °C in PBS. Spectra were measured between 300 and 500 nm using a PerkinElmer LS 55 spectrofluorometer (Waltham, MA, USA) equipped with a thermostated cell holder attached to a Haake F8 water bath (Karlsruhe, Germany). The excitation wavelength was 280 nm. A2  ×  10 mm quartz cuvette was used.

### 2.12. Fibrin Structure Determination by Stimulated Emission Depletion (STED) Super-Resolution and Confocal Microscopy

Fibrin clots prepared as described above were analyzed by stimulated emission depletion (STED) and confocal microscopy. To each reaction (in duplicate), 240 μL of fibrinogen (1 mg/mL) in 100 mmol/L Tris/HCl, 5 mmol/L CaCl_2_, pH 7.4 was seeded on glass cover lips. The polymerization reaction was started by adding 60 μL of thrombin (at a final concentration of 0.25 U/mL) at 25 °C. After 90 min, samples were stained with anti-fibrinogen β-chain (1:100) primary antibody produced in rabbit (HPA001900, Sigma-Aldrich, St. Louis, MO, USA) and Alexa Fluor 555-goat anti rabbit IgG (H + L) secondary antibody (1:500, Life Technologies, Carlsbad, CA, USA). Glycerol was used as mounting medium. STED xyz images (i.e., z-stacks acquired along 3 directions: x, y, and z axes) were acquired by using an SP8 STED 3× confocal microscope (Leica). Alexa Fluor 555 was excited with a 555 nm white light laser and emission collected from 580 to 620 nm. A 660 nm pulsed-depletion laser was used for quenching the fluorescence from molecules at the periphery of the excitation focus. Images were acquired with Leica HC PL APO CS2 100x/1.40 oil STED White objective. Collected images were analyzed with Leica Application Suite X (LAS X) software. No deconvolution was applied.

Fibrin structure was also analyzed by standard confocal microscopy using a Leica plan apo 63× oil immersion objective.

### 2.13. In Vitro Assay: AAPH-Treated Fibrinogen

Increasing concentrations (1–4 mM) of AAPH were incubated with 1 mg of purified human fibrinogen (Sigma, Milan, Italy) dissolved in 1 mL phosphate buffered saline pH 7.4. Samples were incubated at 37 °C for 6 h. To eliminate any AAPH residues in the samples, fibrinogen was recovered and dialyzed against PBS before the assay was conducted. To evaluate the potential preventive effect of an antioxidant on the afore-mentioned AAPH-induced oxidation reaction, 1 mg of purified human fibrinogen (Sigma, Milan, Italy) dissolved in 1 mL phosphate buffered saline pH 7.4 was incubated with 4 mM AAPH in the presence of 0.1mM Trolox at 37 °C for 6 h. Then, fibrinogen was recovered and dialyzed against PBS.

### 2.14. Statistical Analysis

All the experiments were performed in triplicate at 3 different time-points. For each timepoint, the mean value of the three replicates was calculated for each subject. The group median and interquartile range were calculated considering the mean values for each subject as single value in the calculations. The Wilcoxon signed-rank test was applied for all multiple-comparison analysis except for fiber diameter and clot porosity data, which were analyzed by one-way ANOVA (the normality of the variable distribution was checked with the Shapiro–Wilk test). All statistical operations data were processed using the Graph Pad Prism 5 software. A value of *p* < 0.05 was considered as statistically significant.

## 3. Results

### 3.1. Subjects

There were 15 patients, 5 for each Child–Turcotte–Pugh (CTP) class. Demographic and clinical characteristics of the study population are summarized in Table 1. As expected, from subgroup A to subgroup C, there was a progressive worsening of liver function tests comprising coagulation markers and fibrinogen. MELD score also gradually increased from the first to the third subgroup.

### 3.2. Plasma Oxidative Stress Markers in Patients and Controls

**Figure 1:**
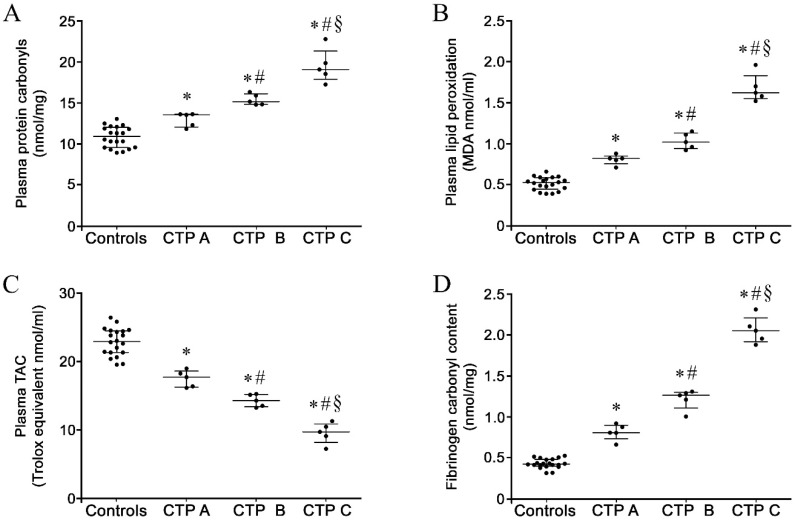
Plasma protein carbonyl content (**A**), plasma lipid peroxidation (**B**), plasma total antioxidant capacity (**C**) and fibrinogen oxidation in purified fibrinogen fractions (**D**) in patients with liver cirrhosis at different disease severity (*n* = 5 for each Child–Turcotte–Pugh category) and controls (*n* = 20). All experiments were performed in triplicate. Values are represented as median with interquartile range. * Significant difference vs. controls at the *p* < 0.05 level. # Significant difference vs. CTP A at the *p* < 0.05 level. § Significant difference vs. CTP B at the *p* < 0.05 level.

As reported in Figure 1, plasma from each patient with liver cirrhosis displayed significantly higher total PC (Figure 1A) and MDA levels (Figure 1B), together with lower total antioxidant capacity (TAC) (Figure 1C) compared to healthy controls. In particular, the levels of the above redox parameters were progressively increased along with disease severity (Child–Turcotte–Pugh score, Figure 1A–C).

### 3.3. Oxidation Levels in Fibrinogen Purified from Patients and Controls

Fibrinogen purified from patients with liver cirrhosis displayed significantly increased carbonylation in comparison to healthy controls, as reported in Figure 1D.

### 3.4. Fibrin Susceptibility to Plasmin-Induced Lysis

**Figure 2:**
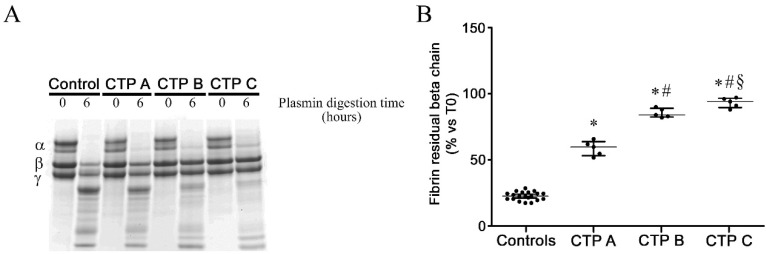
Fibrin resistance to plasmin-induced lysis experiments. (**A**) Representative gel of fibrin degradation after 0, and 6 h of plasmin digestion using fibrinogen purified from patients with liver cirrhosis at different disease severity (*n* = 5 for each Child–Turcotte–Pugh category) and controls (*n* = 20). (**B**) Residual fibrin β chain intensity after 6 h of plasmin digestion in fibrinogen purified from patients with liver cirrhosis at different stages (*n* = 5) and controls (*n* = 20). All experiments were performed in triplicate. Values are represented as median with interquartile range. * Significant difference vs. controls at the *p* < 0.05 level. # Significant difference vs. CTP A at the *p* < 0.05 level. § Significant difference vs. CTP B at the *p* < 0.05 level.

We measured fibrin β chain degradation after plasmin digestion in patients with cirrhosis and in controls (Figure 2A). In cirrhotic patients, the relative band intensity at each considered time of plasmin digestion (Figure 2B) was significantly higher than controls. Interestingly, reduced susceptibility to plasmin-induced lysis was associated with disease severity (Child–Turcotte–Pugh score).

### 3.5. Fibrinogen Polymerization and Fibrin Formation

**Figure 3:**
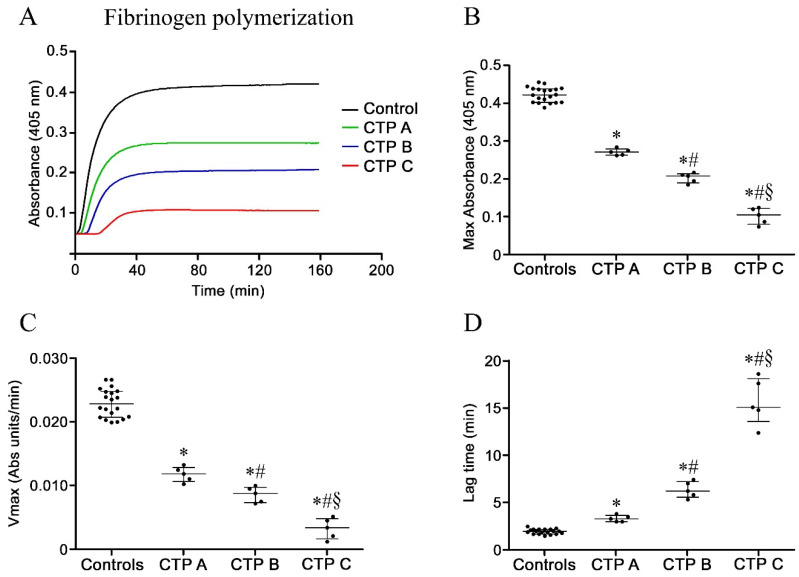
Fibrinogen polymerization experiments. (**A**) Representative curves of thrombin-catalyzed fibrinogen polymerization and corresponding (**B**) Max Absorbance, (**C**) Vmax, and (**D**) lag time in fibrinogen purified from patients with liver cirrhosis at different disease severity (*n* = 5 for each Child–Turcotte–Pugh category) and controls (*n* = 20). Values are represented as median with interquartile range. * Significant difference vs. controls at the *p* < 0.05 level. # Significant difference vs. CTP A at the *p* < 0.05 level. § Significant difference vs. CTP B at the *p* < 0.05 level.

The kinetics of fibrinogen polymerization, as an index of its clotting function, was assessed. Representative curves of thrombin-catalyzed fibrinogen polymerization are shown in Figure 3A. As previously shown, the initial formation of half-staggered, double-stranded protofibrils occurs during a lag phase, in which no turbidity increase is detected; then, lateral aggregation of protofibrils causes an increase in turbidity [22,23]. In patients with cirrhosis, the ability of fibrinogen to undergo polymerization was diminished, as indicated by significant changes in Max Abs, Vmax and lag time (Figure 3B–D), suggesting a different clot structure. All these changes were more marked in patients with more severe disease.

### 3.6. Circular Dichroism Spectra: Analysis of Secondary Structure

**Figure 4:**
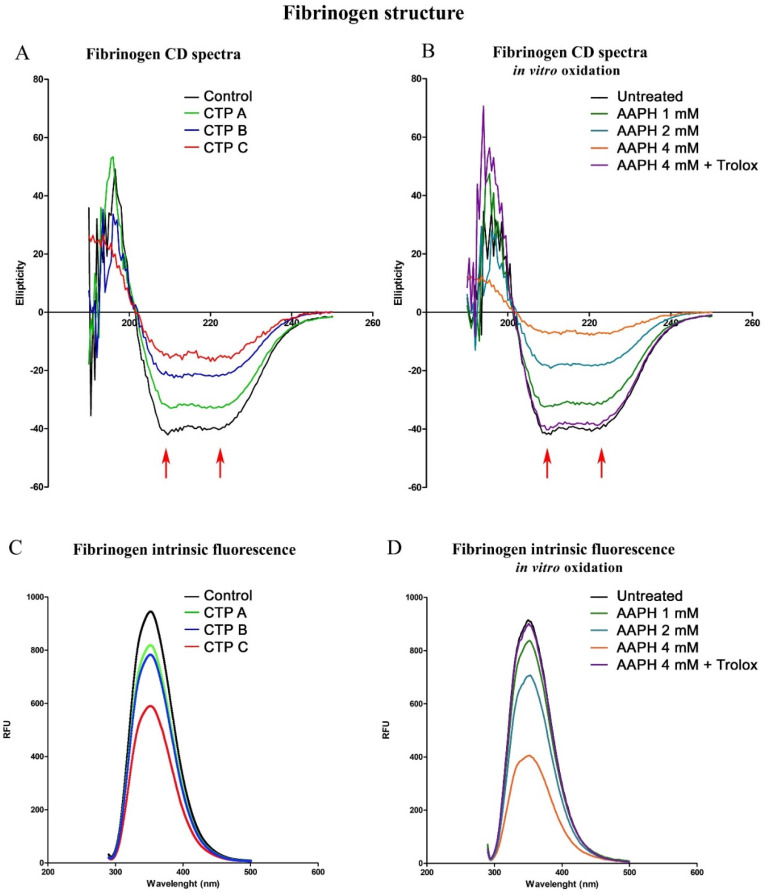
Fibrinogen structure. (**A**) Representative far-UV circular dichroism spectra of fibrinogen purified from patients with liver cirrhosis at different disease severity and controls. The two negative peaks observed in controls at 208 and 222 nm (arrows) are typical of protein α-helix structure. Fibrinogen purified from cirrhotic patients displayed an altered CD spectrum consisting mainly of a decrease in the negative peak in the 215–225 nm region, suggesting a decrease in alpha-helical content. This secondary structure alteration is associated with disease severity. (**B**) To demonstrate whether oxidation could induce fibrinogen secondary structure alterations, in vitro fibrinogen oxidation experiments were performed. After fibrinogen oxidation, the fibrinogen CD spectrum showed an increased ellipticity in an oxidation-dependent manner. Moreover, the antioxidant Trolox was able to prevent these fibrinogen structural changes, demonstrating the key role of oxidation in fibrinogen secondary structure modification. (**C**) Fibrinogen tertiary structure was investigated by intrinsic emission fluorescence spectroscopy. Tryptophan residues that are buried in the core of the protein show high intrinsic fluorescence, as in fibrinogen purified from controls. On the contrary, fibrinogen purified from patients with liver cirrhosis exhibited lower intrinsic fluorescence intensity, indicating changes in protein tertiary structure. (**D**) In line with the CD spectra experiments, in vitro fibrinogen oxidation demonstrated the direct role of tertiary structure alterations. Once again, Trolox treatment prevented these structural changes, demonstrating the pivotal role of oxidation in fibrinogen tertiary structure alterations. The arrows indicate the two negative peaks at 208 and 222 nm, typical of protein α-helix structure.

Secondary protein structure was analyzed by far-UV circular dichroism (CD) spectroscopy (Figure 4A). CD spectrum is a first-class method for secondary structure determination and results from electronic transition between molecular orbitals in ground and excited states of proteins [24]. The two negative peaks at 208 and 222 nm (arrows in Figure 4A) are typical of protein α-helix structure. In controls, the fibrinogen spectrum was indicative of a typically alpha-helical structure with minima at 208 nm and at 222 nm. An altered CD spectrum, mainly consisting of a decrease in the negative peak in the 215–225 nm region, was observed in fibrinogen from cirrhotic patients, suggesting a decrease in alpha-helical content (Figure 4A). Interestingly, this secondary structure alteration was associated with disease severity.

To demonstrate the oxidation-induced fibrinogen structural changes, fibrinogen from healthy subjects was incubated with increasing AAPH concentrations. Fibrinogen CD spectra showed an increased ellipticity at each considered AAPH concentration in an oxidation-dependent manner. To confirm the oxidation-dependent fibrinogen secondary structure alteration, 0.1 mM Trolox was added to AAPH incubation reactions and CD spectrum was then assessed. As shown in Figure 4B, the simultaneous incubation of AAPH and Trolox was able to prevent the observed changes in fibrinogen secondary structure, demonstrating the key role of oxidation in fibrinogen secondary structure modification.

### 3.7. Intrinsic Fluorescence Spectroscopy Analysis

Intrinsic emission fluorescence spectroscopy can be used to investigate changes in protein tertiary structure and to estimate their microenvironment and the amount of present tryptophan residues. Tryptophan residues are usually buried in the core of the protein, having high intrinsic fluorescence, typically between 331 and 347 nm [25]. Fibrinogen from patients with liver cirrhosis showed lower intrinsic fluorescence intensity values than fibrinogen purified from controls, indicating changes in protein tertiary structure (Figure 4C). These changes were very marked in fibrinogen purified from CTP C patients. Moreover, similar to data obtained with CD spectra, fibrinogen intrinsic emission fluorescence showed decreased values in an oxidation-dependent manner (Figure 4D), and Trolox treatment prevented these changes, demonstrating the key role of oxidation in fibrinogen tertiary structure modification.

### 3.8. Confocal Microscopy Analysis

**Figure 5:**
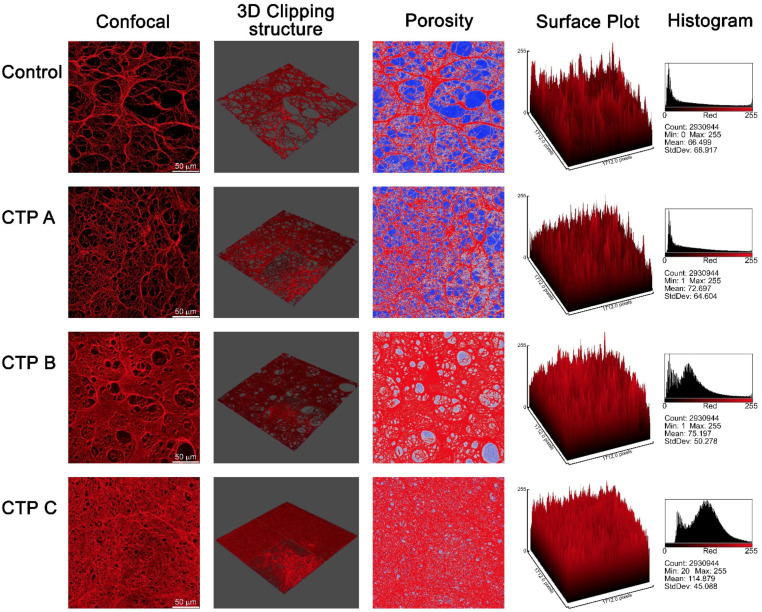
Confocal microscopy analysis of fibrin gels of fibrinogen purified from patients with liver cirrhosis at different stages and controls. Three-dimensional confocal microscopy images clearly show control fibrin gel characterized by large pores and tick fibers when compared to fibrin from cirrhotic patients which are much dense, with narrow pores and thin fibers. In particular, fibrin gels from fibrinogen purified from CTP B and CTP C patients exhibited a dramatic gel rearrangement: fibers were so closely packed in the bulk of gel, obscuring the individual fibers and producing thin sheets with small pores. Surface plot and histogram values are referred to in the corresponding confocal image (first column).

Fibrin gels fall into two main categories, one represented by thin gels composed of a small number of thin fibers which form a semiflexible polymer network characterized by narrow pores, and the second made of a great number of thick, much stiffer long fibers with show large pores. Three-dimensional confocal microscopy images (Figure 5) show the network density and gel porosity of fibrin clots from patients with cirrhosis and controls. Fibrinogen purified from patients produced markedly different fibrin networks when compared to those purified from healthy subjects, mainly in terms of pore size and clot porosity, which appeared both strongly reduced. The fibrin clot from CTP A patients revealed densely packed thin fibers and smaller pores when compared to controls, albeit maintaining a similar structure. In contrast, fibrin gels from CTP B and CTP C patients exhibited a dramatic gel rearrangement, with fibers closely packed in the bulk of gel obscuring the individual fibers and producing thin sheets with small pores. This peculiar structure was even more marked in CTP C patients, where fibrin formed a homogeneous dense film with very small pores.

### 3.9. STED Super-Resolution Microscopy Analysis

**Figure 6:**
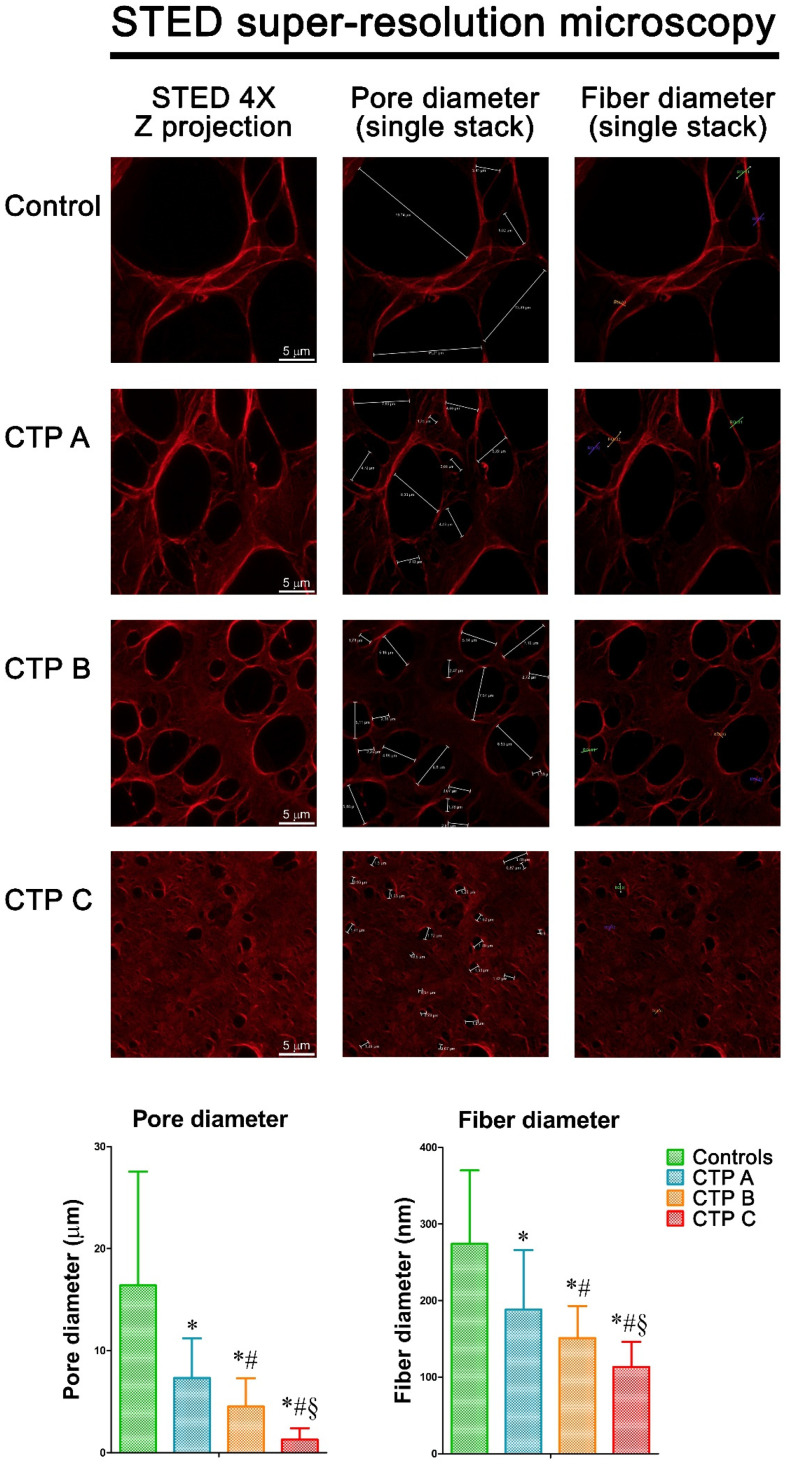
STED super-resolution microscopy analysis of fibrin gels of fibrinogen purified from patients with liver cirrhosis at different disease severity (*n* = 5 for each Child–Turcotte–Pugh category) and controls. STED super-resolved microscopy revealed a marked increase in fiber density and clot porosity in fibrin from cirrhotic patient when compared to controls. Leica Application Suite X Software analysis demonstrated that fibrin fibers from fibrinogen purified from cirrhotic patients showed a significant decrease in fiber diameter and clot porosity when compared to controls, and this became more marked with disease progression. Values are represented as mean ± SD. * Significant difference vs. controls at the *p* < 0.05 level. # Significant difference vs. CTP A at the *p* < 0.05 level. § Significant difference vs. CTP B at the *p* < 0.05 level.

This is the first report of fibrin ultrastructure analysis by this innovative technique. STED super-resolved microscopy provides precise details about fibrin network both in terms of individual fibers and pore diameter. Using fibrin fibers from fibrinogen purified from patients and controls, STED super-resolved microscopy revealed marked alterations in fiber diameter and in clot porosity in cirrhotic patients when compared to controls (Figure 6). In particular, fibrin analysis indicated that fiber diameter in samples from cirrhotic patients was significantly decreased in comparison to controls, and this became more marked with disease progression. Moreover, fibrin from controls was characterized by the presence of large pores whose size decreased when disease severity increased (Figure 6).

## 4. Discussion

In the present pilot study, STED super-resolution microscopy revealed the presence of marked alteration in clot porosity and fibrin fiber diameter in fibrinogen fractions purified from cirrhotic patients compared to fibrin from controls. These findings should be viewed in the complex picture of the fragile hemostatic balance of cirrhotic patients [1,26] which are characterized by reduced synthesis of both procoagulant and anticoagulant proteins, but also by high plasma levels of von Willebrand factor and factor VIII and, consequently, by thrombin hyperactivation [27]. Fibrinogen strictly regulates clot formation rate, structure and mechanical and fibrinolytic stability. Hence, qualitative and quantitative alterations in fibrinogen and fibrin levels represent main determinants for thrombosis risk [12,14,15,28,29,30,31], and could be implicated in the increase in prothrombotic risk observed in cirrhotic patients.

Interestingly, among plasma proteins, fibrinogen is the major target of reactive oxygen species (ROS) which are responsible for structural and functional modifications [32,33], mainly due to carbonyl group formation, hydrogen ion abstraction, and protein–protein cross-linkages [34]. In line with other studies showing redox imbalance in patients with liver cirrhosis [35,36], our data indicate marked oxidative post-translational fibrinogen modifications in these patients. As previously reported, oxidative alterations can have a deep impact on fibrinogen function, ultimately producing prothrombotic clots [15,16]. The observed alterations are associated with disease severity, in line with the results of Hugenholtz and collaborators [37]. However, these authors did not find any alteration in fibrinogen polymerization kinetic parameters such as Vmax and Max Abs, but only a slight increase in lag time—restricted to CTP B patients. Furthermore, in their study, neither fibrin density nor fiber diameter modifications in cirrhotic patients when compared to healthy volunteers were observed and reduced clot permeability was attributed to excessive fibrinogen carbonylation. The study presented herein provides new and relevant findings about fibrinogen protein structure, fibrin susceptibility to lysis and super-resolution microscopy, contributing to elucidating, in a mechanistic fashion, thrombus formation in cirrhosis.

Fibrin polymerization kinetic assays—where kinetic parameters were associated with fibrinogen carbonyl content—suggest a key role of oxidation on fibrin polymerization. Accordingly, it has been shown that upon carbonyl group addition, polypeptide chain conformation results were markedly altered with consequent functional activity alterations [38]. Moreover, the presence of amino acids, such as proline and arginine (which are highly susceptible to oxidation), in the cleavage site of fibrinogen by thrombin, may explain, at least in part, the altered parameters characterizing fibrin polymerization kinetic in patients with cirrhosis [39].

Fibrinogen secondary structure analysis by far-UV circular dichroism spectroscopy revealed, in cirrhotic patients, a decrease in α-helix content, which was strictly associated with disease severity. Taking into account that protein structure defines protein function, our in vitro data confirm that fibrinogen carbonylation promotes fibrinogen secondary structure alterations, affecting the biological function of fibrinogen. These data were also confirmed by fibrinogen tertiary structure analysis obtained by intrinsic fluorescence spectra. In addition, antioxidant (Trolox, a cell permeable, water-soluble vitamin E analog) treatment reverted the oxidation-dependent structure modification, confirming the direct role of oxidation in the alteration of fibrinogen structure.

Fibrin from cirrhotic patients was characterized by resistance to lysis when compared to fibrinogen purified from controls, and also this parameter was associated with disease severity. Moreover, in line with our previous data [15,16], fibrin resistance to lysis correlated with fibrinogen oxidation levels in patients with liver cirrhosis, suggesting a direct role of oxidation on fibrin degradation. This important finding was in agreement with a recent report that demonstrates the association between fibrin clot structure and thrombotic risk in cirrhosis [37]. The present data are in agreement with previous findings, revealing that abnormal fibrin polymerization leads to the formation of altered fibrin networks associated with a prothrombotic phenotype [40]. Furthermore, those changes in fibrin structure directly affect the rate of fibrinolysis [41,42].

Many pathologic conditions are associated with an abnormal fibrin network, even if the mechanisms underlying these structural changes remain unknown. Clinical studies have demonstrated that plasma clots from thrombotic diseases, such as ischemic stroke, diabetes and VTE, are dense and composed of thin fibrin fibers with reduced clot porosity and delayed fibrinolysis when compared to controls [29,43,44]. Strikingly, fibrin structure measurements have been suggested as thrombosis biomarkers [45]. In the present study, super-resolution STED confocal microscopy demonstrated, for the first time, oxidative stress-induced clot ultrastructure modification in patients with liver cirrhosis. Fibrin clot from cirrhotic patients appeared composed of densely packed fibers, less susceptible to lysis. This structural change is consistent with the already proposed observation that thinner fibers are denser than thicker ones, hence suggesting that molecule packing increases with the decreasing of fiber diameter. It should be underscored that with the increase in compact, highly branched networks with thin fibers significantly correlated with cirrhosis severity. Clots composed of thin fibers and small pores have been suggested to be more thrombogenic [42,46,47], but the mechanisms underlying the formation of these prothrombotic fibrin clots have not yet been described. Our in vitro experiments revealed that oxidative stress is a strong determinant of these evident clot structural changes.

Several limitations of this study should be acknowledged. First, a small number of patients was included, and larger studies will be needed to demonstrate associations with fibrinogen oxidation and thrombotic events in cirrhosis. In regard to the few number of enrolled patients, we are planning to increase it in the future. However, our very innovative findings are considerably sound and significant, despite the fact that they were acquired via a pilot study. Moreover, the number of patients did not allow the estimation of differences among the etiologies of chronic liver disease. Interestingly, it is well known that alcohol strongly and actively alters oxidative status [48]. Indeed, we included only patients with a protracted alcohol abstinence (at least 6 months). It would be interesting to develop further studies aimed to specifically analyze other main causes of cirrhosis, such as hepatitis C virus (HCV) infection. Second, genetic polymorphisms known to modify the fibrin structure were not investigated. Moreover, γ’ fibrinogen—arising from alternative mRNA processing—was not analyzed. It has been shown that higher plasma concentrations of γ’ fibrinogen yield thrombi that are very resistant to fibrinolysis [49,50]. Nonetheless, our data clearly demonstrate that oxidative-mediated fibrinogen structure modifications such as those observed in cirrhotic patients are associated with thrombosis tendencies and disease severity, suggesting new potential targets for innovative therapeutic approaches. Another limitation consists in the lack of long-term clinical outcomes such as development of portal vein thrombosis (PVT). However, coherently with our data, the severity of liver disease can be correlated with the risk of PVT [51]. Regarding the possible clinical implications of the present study, it is well known that thrombotic events such as PVT are relatively common in cirrhotic patients, with a 5-year incidence of 10–20% [52,53].

In conclusion, the results of the present study show that in patients with liver cirrhosis (a) a systemic oxidative stress is associated with an increased level of fibrinogen oxidation; (b) fibrinogen structural alterations are present and associated with fibrinogen oxidation levels; (c) the increased extent of fibrinogen structural changes is associated with altered fibrinogen polymerization and fibrin degradation; (d) STED super-resolution microscopy reveals, for the first time, fibrin clot ultrastructure modifications, highlighting the direct impact of oxidative stress in clot porosity and fibrin fiber diameter; (e) all these changes correlate with disease severity. Finally, (f) our in vitro experiments clearly confirm the direct role of oxidation on fibrinogen structural modification.

## Figures and Tables

**Table 1:** Clinical and laboratory patterns of the study population stratified by Child–Turcotte–Pugh class.

	Healthy Controls (*n* = 20)	CTP A (*n* = 5)	CTP B (*n* = 5)	CTP C (*n* = 5)
**Sex (M/F)**	10/10	2/3	3/2	3/2
**Age (mean ± SD years)**	65 ± 7	65 ± 13	68 ± 8	62 ± 9
**Etiology**				
HCV	/	2	2	3
Alcohol	/	3	1	0
NASH	/	0	1	1
Other	/	0	1	1
**Ascites**				
absent	/	2	0	0
mild	/	3	3	1
moderate	/	0	2	3
severe	/	0	0	1
**HE**				
Grade 0	/	3	1	0
Grade 1	/	2	1	1
Grade 2	/	0	3	4
Grade 3	/	0	0	0
Grade 4	/	0	0	0
**Total bilirubin (M (range) mg/dL)**	/	0.8 (0.5–2)	1.2 (1–2)	4.9 (2.3–7.2)
**Albumin (M (range) g/dL)**	/	3.7 (3.5–4.7)	3.4 (2.5–3.6)	3.3 (2.1–3.7)
**INR**	1 (0.8–1.2)	1.4 (1–1.5)	1.3 (1.1–1.4)	1.7 (1.2–2.2)
**Hemoglobin (M (range) g/dL)**	13.2 (11.5–16.4)	11.2 (10.7–12)	10.2 (8.1–12.7)	9.9 (8.1–13.4)
**Hematocrit (%)**	/	34.0 (33.1–39)	32 (25.2–38)	28.7 (23.3–37)
**Creatinine (M (range) mg/dL)**	/	0.8 (0.5–0.8)	1 (0.6–1.8)	0.7 (0.5–0.8)
**Sodium (M (range) mmol/L)**	/	138.5 (136–145)	136.5 (131–144)	136 (128–145)
**Potassium (M (range) mmol/L)**	/	4.1 (3.9–4.3)	4.1 (3.4–5.1)	4.2 (3.8–4.5)
**Calcium (M (range) mg/dL)**	/	8.6 (8.1–8.8)	7.9 (7.4–8.7)	8.1 (7.5–9.1)
**Prothrombin time (M (range) s)**	/	6.7 (6–7.4)	6.2 (5–7.2)	5.8 (5–6.8)
**Fibrinogen (M (range) mg/dL)**	295 (233–392)	369.5 (315–544)	286 (162–327)	188 (145–247)
**MELD (mean ± SD)**	/	10 ± 1	11 ± 3	18 ± 3

CTP, Child–Turcotte–Pugh; M, males; F, females; χ2, chi square test; MW-U, Mann–Whitney U test; HCV, hepatitis C virus; NASH, non-alcoholic steatohepatitis; SD, standard deviation; ns, not significant (*p* > 0.05); HE, hepatic encephalopathy (classified by West Haven criteria); M, median; INR, international normalized ratio; MELD, model for end-stage liver disease.

## References

[1] Turco L., De Raucourt E., Valla D., Villa E. (2019). Anticoagulation in the cirrhotic patient. JHEP Rep..

[2] Caldwell S.H., Hoffman M., Lisman T., Macik B.G., Northup P., Reddy K.R., Tripodi A., Sanyal A.J. (2006). Coagulation in Liver Disease Group Coagulation disorders and hemostasis in liver disease: Pathophysiology and critical assessment of current management. Hepatology.

[3] Tripodi A., Mannucci P.M. (2007). Abnormalities of hemostasis in chronic liver disease: Reappraisal of their clinical significance and need for clinical and laboratory research. J. Hepatol..

[4] Talving P., Lustenberger T., Okoye O., Lam L., Smith J.A., Inaba K., Mohseni S., Chan L., Demetriades D. (2013). The impact of liver cirrhosis on outcomes in trauma patients. J. Trauma Acute Care Surg..

[5] Gulley D., Teal E., Suvannasankha A., Chalasani N., Liangpunsakul S. (2008). Deep Vein Thrombosis and Pulmonary Embolism in Cirrhosis Patients. Dig. Dis. Sci..

[6] Ambrosino P., Tarantino L., Di Minno G., Paternoster M., Graziano V., Petitto M., Nasto A., Di Minno M.N.D., Di Minno M.N., Di Minno M.N.D. (2017). The risk of venous thromboembolism in patients with cirrhosis. Thromb. Haemost..

[7] Tripodi A., Mannucci P.M. (2011). The Coagulopathy of Chronic Liver Disease. N. Engl. J. Med..

[8] Sipeki N., Antal-Szalmas P., Lakatos P., Papp M. (2014). Immune dysfunction in cirrhosis. World J. Gastroenterol..

[9] Lados-Krupa A., Konieczynska M., Chmiel A., Undas A. (2015). Increased Oxidation as an Additional Mechanism Underlying Reduced Clot Permeability and Impaired Fibrinolysis in Type 2 Diabetes. J. Diabetes Res..

[10] Helms C.C., Kapadia S., Gilmore A.C., Lu Z., Basu S., Kim-Shapiro D.B. (2017). Exposure of fibrinogen and thrombin to nitric oxide donor ProliNONOate affects fibrin clot properties. Blood Coagul. Fibrinolysis.

[11] Marí M., Colell A., Morales A., Von Montfort C., García-Ruiz C., Fernández-Checa J.C. (2010). Redox Control of Liver Function in Health and Disease. Antioxid. Redox Signal..

[12] Becatti M., Emmi G., Bettiol A., Silvestri E., Di Scala G., Taddei N., Prisco M., Fiorillo C. (2018). Behçet’s syndrome as a tool to dissect the mechanisms of thrombo-inflammation: Clinical and pathogenetic aspects. Clin. Exp. Immunol..

[13] Lami D., Cellai A.P., Antonucci E., Fiorillo C., Becatti M., Grifoni E., Cenci C., Marcucci R., Mannini L., Miniati M. (2014). Residual perfusion defects in patients with pulmonary embolism are related to impaired fibrinolytic capacity. Thromb. Res..

[14] Miniati M., Fiorillo C., Becatti M., Monti S., Bottai M., Marini C., Grifoni E., Formichi B., Bauleo C., Arcangeli C. (2010). Fibrin Resistance to Lysis in Patients with Pulmonary Hypertension Other than Thromboembolic. Am. J. Respir. Crit. Care Med..

[15] Becatti M., Marcucci R., Bruschi G., Taddei N., Bani D., Gori A.M., Giusti B., Gensini G.F., Abbate R., Fiorillo C. (2014). Oxidative Modification of Fibrinogen Is Associated with Altered Function and Structure in the Subacute Phase of Myocardial Infarction. Arter. Thromb. Vasc. Boil..

[16] Becatti M., Emmi G., Silvestri E., Bruschi G., Ciucciarelli L., Squatrito D., Vaglio A., Taddei N., Abbate R., Emmi L. (2016). Neutrophil Activation Promotes Fibrinogen Oxidation and Thrombus Formation in Behçet Disease. Circulation.

[17] Pugh R.N.H., Murray-Lyon I.M., Dawson J.L., Pietroni M.C., Williams R. (1973). Transection of the oesophagus for bleeding oesophageal varices. Br. J. Surg..

[18] Bernardi M., Gitto S., Biselli M. (2011). The MELD score in patients awaiting liver transplant: Strengths and weaknesses. J. Hepatol..

[19] Sofi F., Dinu M., Pagliai G., Cesari F., Gori A.M., Sereni A., Becatti M., Fiorillo C., Marcucci R., Casini A. (2018). Low-Calorie Vegetarian Versus Mediterranean Diets for Reducing Body Weight and Improving Cardiovascular Risk Profile. Circulation.

[20] Fiorillo C., Becatti M., Attanasio M., Lucarini L., Nassi N., Evangelisti L., Porciani M.C., Nassi P., Gensini G., Abbate R. (2010). Evidence for oxidative stress in plasma of patients with Marfan syndrome. Int. J. Cardiol..

[21] Munishkina L.A., Phelan C., Uversky V.N., Fink A.L. (2003). Conformational Behavior and Aggregation of α-Synuclein in Organic Solvents: Modeling the Effects of Membranes†. Biochemistry.

[22] Wolberg A.S., Gabriel D.A., Hoffman M. (2002). Analyzing fibrin clot structure using a microplate reader. Blood Coagul. Fibrinolysis.

[23] Weisel J., Nagaswami C. (1992). Computer modeling of fibrin polymerization kinetics correlated with electron microscope and turbidity observations: Clot structure and assembly are kinetically controlled. Biophys. J..

[24] Greenfield N.J. (2006). Using circular dichroism spectra to estimate protein secondary structure. Nat. Protoc..

[25] Hellmann N., Schneider D. (2019). Hands On: Using Tryptophan Fluorescence Spectroscopy to Study Protein Structure. Breast Cancer.

[26] Lisman T., Porte R.J. (2010). Rebalanced hemostasis in patients with liver disease: Evidence and clinical consequences. Blood.

[27] Hollestelle M.J., Geertzen H.G.M., Straatsburg I.H., Van Gulik T.M., Van Mourik J.A. (2004). Factor VIII expression in liver disease. Thromb. Haemost..

[28] Collet J.-P., Allali Y., Lesty C., Tanguy M., Silvain J., Ankri A., Blanchet B., Dumaine R., Gianetti J., Payot L. (2006). Altered Fibrin Architecture Is Associated with Hypofibrinolysis and Premature Coronary Atherothrombosis. Arter. Thromb. Vasc. Boil..

[29] Undas A., Zawilska K., Ciesla-Dul M., Lehmann-Kopydłowska A., Skubiszak A., Ciepłuch K., Tracz W. (2009). Altered fibrin clot structure/function in patients with idiopathic venous thromboembolism and in their relatives. Blood.

[30] Dunn E.J., Ariëns R.A., Grant P.J. (2005). The influence of type 2 diabetes on fibrin structure and function. Diabetologia.

[31] Undas A., Ariëns R.A. (2011). Fibrin Clot Structure and Function. Arter. Thromb. Vasc. Boil..

[32] Karlaftis V., Perera S., Monagle P., Ignjatovic V. (2016). Importance of post-translational modifications on the function of key haemostatic proteins. Blood Coagul. Fibrinolysis.

[33] Shacter E., Williams J.A., Lim M., Levine R.L. (1994). Differential susceptibility of plasma proteins to oxidative modification: Examination by western blot immunoassay. Free. Radic. Boil. Med..

[34] Stadtman E.R., Levine R.L. (2003). Free radical-mediated oxidation of free amino acids and amino acid residues in proteins. Amino Acids.

[35] Li S., Tan H.-Y., Wang N., Zhang Z.-J., Lao L., Wong C.-W., Feng Y. (2015). The Role of Oxidative Stress and Antioxidants in Liver Diseases. Int. J. Mol. Sci..

[36] Vairappan B. (2015). Endothelial dysfunction in cirrhosis: Role of inflammation and oxidative stress. World J. Hepatol..

[37] Hugenholtz G.C., Macrae F.L., Adelmeijer J., Dulfer S., Porte R.J., Lisman T., Ariëns R.A. (2016). Procoagulant changes in fibrin clot structure in patients with cirrhosis are associated with oxidative modifications of fibrinogen. J. Thromb. Haemost..

[38] Cai Z., Yan L.-J. (2013). Protein Oxidative Modifications: Beneficial Roles in Disease and Health. J. Biochem. Pharmacol. Res..

[39] Gallwitz M., Enoksson M., Thorpe M., Hellman L.T. (2012). The Extended Cleavage Specificity of Human Thrombin. PLoS ONE.

[40] Siudut J., Grela M., Wypasek E., Plens K., Undas A. (2016). Reduced plasma fibrin clot permeability and susceptibility to lysis are associated with increased risk of postthrombotic syndrome. J. Thromb. Haemost..

[41] Weisel J.W., Litvinov R.I. (2013). Mechanisms of fibrin polymerization and clinical implications. Blood.

[42] Weisel J.W. (2007). Structure of fibrin: Impact on clot stability. J. Thromb. Haemost..

[43] Bridge K., Philippou H., Ariëns R.A. (2014). Clot properties and cardiovascular disease. Thromb. Haemost..

[44] Colle J.P., Mishal Z., Lesty C., Mirshahi M., Peyne J., Baumelou A., Bensman A., Soria J., Soria C. (1999). Abnormal fibrin clot architecture in nephrotic patients is related to hypofibrinolysis: Influence of plasma biochemical modifications: A possible mechanism for the high thrombotic tendency?. Thromb. Haemost..

[45] Ariëns R.A. (2013). Fibrin(ogen) and thrombotic disease. J. Thromb. Haemost..

[46] Undas A. (2017). Prothrombotic Fibrin Clot Phenotype in Patients with Deep Vein Thrombosis and Pulmonary Embolism: A New Risk Factor for Recurrence. BioMed Res. Int..

[47] Weisel J.W., Litvinov R.I. (2017). Fibrin Formation, Structure and Properties. Sub Cell. Biochem..

[48] Addolorato G., Abenavoli L., Dallio M., Federico A., Germani G., Gitto S., Leandro G., Loguercio C., Marra F., Stasi E. (2020). Alcohol associated liver disease 2020: A clinical practice guideline by the Italian Association for the Study of the Liver (AISF). Dig. Liver Dis..

[49] Lovely R.S., Kazmierczak S.C., Massaro J.M., D’Agostino R.B., O’Donnell C.J., Farrell D.H. (2010). γ′ Fibrinogen: Evaluation of a New Assay for Study of Associations with Cardiovascular Disease. Clin. Chem..

[50] Appiah D., Schreiner P.J., MacLehose R.F., Folsom A.R. (2015). Association of Plasma γ’ Fibrinogen With Incident Cardiovascular Disease: The Atherosclerosis Risk in Communities (ARIC) Study. Arter. Thromb. Vasc. Boil..

[51] Chen H., Trilok G., Wang F., Qi X., Xiao J., Yang C. (2014). A single hospital study on portal vein thrombosis in cirrhotic patients—clinical characteristics & risk factors. Indian J. Med. Res..

[52] Nery F., Chevret S., Condat B., De Raucourt E., Boudaoud L., Rautou P.-E., Plessier A., Roulot D., Chaffaut C., Bourcier V. (2015). Causes and consequences of portal vein thrombosis in 1243 patients with cirrhosis: Results of a longitudinal study. Hepatology.

[53] Maruyama H., Okugawa H., Takahashi M., Yokosuka O. (2013). De novo Portal Vein Thrombosis in Virus-Related Cirrhosis: Predictive Factors and Long-Term Outcomes. Am. J. Gastroenterol..

